# Drainage of subconjunctival hemorrhage through conjunctival lymphatic pathways

**DOI:** 10.1016/j.ajoc.2025.102368

**Published:** 2025-07-01

**Authors:** Ashton Zheng-Boon Lau, Grace YF. Tang, William H. Morgan, Geoffrey ZP. Chan

**Affiliations:** a Centre for Ophthalmology and Visual Science, The University of Western Australia, Perth, Western Australia, Australia; b Lions Eye Institute, Perth, Western Australia, Australia; c Department of Ophthalmology, Fremantle and Fiona Stanley Fremantle Hospitals Group, Perth, Western Australia, Australia

**Keywords:** Lymphatics, Conjunctiva, Aqueous humor, Subconjunctival hemorrhage, Bleb-forming surgery

## Abstract

**Purpose:**

To demonstrate the role of ocular lymphatic involvement in aiding the drainage and resolution of subconjunctival hemorrhage.

**Methods:**

We present the case of a 63-year-old Caucasian female who developed a subconjunctival hemorrhage following the administration of a subconjunctival anesthetic during cataract surgery. Intraoperative optical coherence tomography was utilized to image these structures within the deep and middle conjunctival-Tenon's space.

**Results:**

Subconjunctival hemorrhage was visualized and saccular blood-filled structures developed adjacent to sites of hemorrhage. Intraoperative optical coherence tomography confirmed the presence of blood within structures containing partitions reminiscent of valvular leaflets, indicating their lymphatic origin. Rapid resolution of the subconjunctival hemorrhage was noted during the immediate postoperative period.

**Conclusion:**

Subconjunctival hemorrhage may have its resolution assisted by conjunctival lymphatic vessels. Our findings confirm that lymphatic structures may play an under-appreciated role in assisting the clearance of macromolecules from the subconjunctival space.

## 1 Introduction

Subconjunctival hemorrhage (SCH) is a common sequalae of ocular trauma or surgery. While SCH is typically a benign and self-resolving condition, there is no universally accepted treatment to accelerate its resolution.^1^ Resolution may be linked to factors such as systemic blood pressure, the extent of initial hemorrhage and the presence of bleeding disorders.^1^

A healthy lymphatic system is critical to healthy physiological tissue function, and its presence within human conjunctival tissue has been identified. Given that lymphatics play a role in interstitial fluid homeostasis and immunosurveillance, it is conceivable that they may assist in the clearance of blood from the conjunctiva, as documented within other central nervous system (CNS) structures.^2^ The role of lymphatic structures in the eye has also been theorized to affect aqueous humor drainage, intraocular pressure regulation, and fluid balance, and it remains a focus of active research.^3^, ^4^, ^5^

We present a case of SCH where conjunctival lymphatic structures are demonstrated to aid in the drainage and clearance of extravasated blood from the subconjunctival space. Intraoperative optical coherence tomography (iOCT) was used to identify and characterize these lymphatic structures. Our patient experienced a rapid resolution of SCH, highlighting a novel role of the conjunctival lymphatic system in accelerating the clearance of blood and macromolecules within the subconjunctival space.

## 2 Methods

A 63-year-old Caucasian female was referred for left cataract surgery. Her ophthalmic background included a previous episode of acute angle-closure glaucoma in her left eye, treated with YAG laser peripheral iridotomy. She had a past medical history of hypertension.

**Fig. 1:**
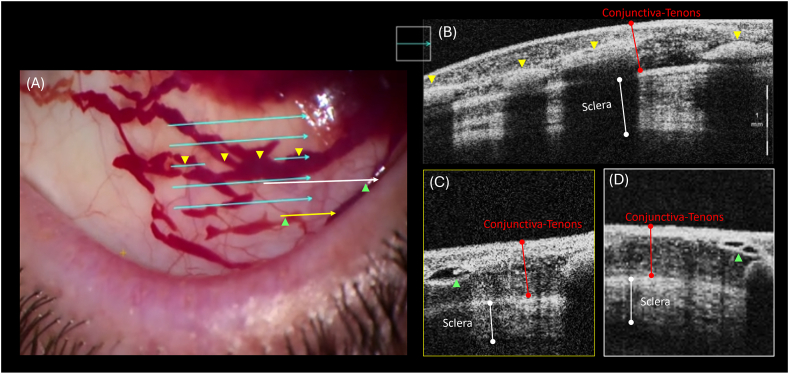
**Intra-operative OCT imaging of the superior bulbar conjunctiva.** (A) Image of the operating microscope view with colored arrows indicating intra-operative OCT scan lines. Magnified inset of OCT scan line corresponding to the middle blue arrow (B) with yellow arrowheads demonstrating the blood-filled sausage shaped structures present within the deep and middle conjunctival-tenons space. Yellow arrow with corresponding OCT inset (C) and white arrow with corresponding inset (D) demonstrate partitions reminiscent of valve leaflets (green arrowheads) present within the non-blood-filled regions of the structures adjacent to the hemorrhage.

Pre-operatively, the patient was dilated with phenylephrine 0.5 %, cyclopentolate 1 %, and atropine 1 % eye drops. Subconjunctival anesthetic was administered in the superior nasal quadrant using a 50:50 mix of 2 % lignocaine and 0.5 % bupivacaine, delivered via a 30-gauge needle. Following anesthetic, SCH developed around the site of injection and within 30 seconds, saccular blood-filled structures were noted to develop, contiguous with the site of SCH. iOCT was completed within the region of these structures utilizing a Zeiss OPMI Lumera 700 microscope (Carl Zeiss Meditec Production LLC) with an integrated OCT function (Fig. 1).

## 3 Results

**Fig. 2:**
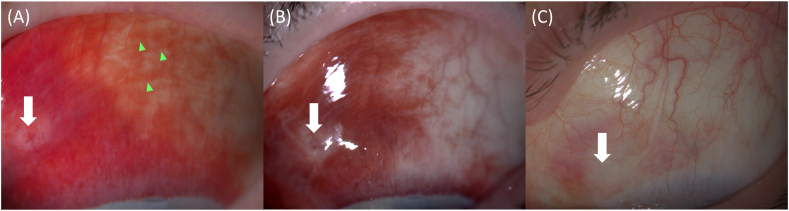
**Slit lamp imaging of subconjunctival hemorrhage.** Rapid resolution of hemorrhage is demonstrated between postoperative day 1 (A) and day 2 (B) with a white arrow denoting the original site of subconjunctival hemorrhage. Blood-filled lymphatic structures are visible during the recovery process (green arrowheads). Further resolution of the SCH and clearing of blood within lymphatic channels is documented at 2 weeks (C).

The patient was reviewed on postoperative day one, day two and two weeks after the procedure to document the resolution of the SCH (Fig. 2). Marked resolution of the SCH was observed between days one and two post-treatment. This was followed by steady improvement over the subsequent 2-week period.

## 4 Discussion

We report a photo-documented case of blood drainage from the subconjunctival space facilitated by conjunctival lymphatic structures in the human eye, opportunistically imaged using intraoperative OCT. Despite identifying multiple risk factors and conditions associated with SCH development and recurrence, the clinical literature lacks positive predictive factors for faster SCH absorption.^1^ The assisted clearance of blood and macromolecules via lymphatic structures within the CNS is well documented, and conjunctival lymphatic function may be a novel factor we have identified in SCH resolution.^2^^,^^6^

Under normal physiological conditions, conjunctival lymphatics are not identifiable due to their thin walls and clear intraluminal fluid, making them a challenge to study. Current methods for imaging conjunctival lymphatics, such as indocyanine green (ICG) channelography and aqueous angiography, rely on the injection of tracers or exogenous dyes.^3^ We combined the opportunistic use of iOCT with the presence of blood used as an endogenous tracer, which highlighted the lymphatic structures, making them amenable for study.

Although blood within the lymphatic structures causes hyperintense signaling on OCT—potentially obscuring the valve-like structures—we have correlated these hyperintense lumens on OCT (Fig. 1B) with the blood-filled, sausage-shaped structures observed under the operating microscope (Fig. 1A), as highlighted by the yellow arrowheads. These structures, which are visible to the naked eye, are more consistent with lymphatic collector channels, which can exceed 200 μm in diameter.^7^ This contrasts with aqueous veins, which have a mean diameter of approximately 50 μm, and conjunctival blood vessels, which have a median diameter of approximately 20 μm.^7^, ^8^, ^9^

We identified lymphatic structures in the conjunctiva by their thin walls, dark lumen, and the distinguishing presence of structures resembling valvular leaflets, which promote the unidirectional movement of lymph or fluid (Fig. 1C and D). Our findings align with previously published descriptions of conjunctival lymphatics.^7^^,^^10^^,^^11^ Additionally, we verified the presence of extravasated erythrocytes within adjacent lymphatic pathways. Our observation of blood drainage away from the site of original SCH parallels the notion that healthy conjunctival lymphatics may assist in fluid clearance from sites of bleb-forming glaucoma surgery. Healthy conjunctival lymphatics have been theorized to improve the outcomes of glaucoma filtration surgery by assisting in the clearance of aqueous and pro-inflammatory mediators from bleb tissue.^3^, ^4^, ^5^ Future studies may elucidate if the presence of healthy conjunctival lymphatics can predict the rapidity of SCH resolution and if there is a relation to the success rates of bleb-forming glaucoma surgery.

## CRediT authorship contribution statement

**Ashton Zheng-Boon Lau:** Writing – review & editing, Writing – original draft. **Grace YF. Tang:** Writing – review & editing, Writing – original draft. **William H. Morgan:** Writing – review & editing. **Geoffrey ZP. Chan:** Methodology, Conceptualization, Writing – review & editing, Investigation.

## Patient consent

Consent to publish this case report has been obtained from the patient in writing, including publication of images.

## Authorship

All authors attest that they meet the current ICMJE criteria for Authorship.

## Declaration of generative AI and AI-assisted technologies in the writing process

During the preparation of this work, the author(s) used ChatGPT3.5 in order to improve the readability and language of the manuscript. After using this service, the author(s) reviewed and edited the content as needed and take full responsibility for the content of the published article.

## Funding

The authors received no funding or grant support for the research, authorship, and/or publication of this article.

## Declaration of competing interest

The authors declare that they have no known competing financial interests or personal relationships that could have appeared to influence the work reported in this paper.
